# DEVELOPING A NOVEL FRAILTY INDEX TO STUDY FRAILTY OF SEXUAL AND GENDER MINORITY OLDER ADULTS IN THE ALL OF US DATABASE

**DOI:** 10.1093/geroni/igac059.2733

**Published:** 2022-12-20

**Authors:** Chelsea Wong, Michael Wilczek, Jordon Bosse, Louisa Smith, Justin Manjourides, Ariela Orkaby, Brianne Olivieri-Mui

**Affiliations:** Harvard Multicampus Geriatric Medicine Fellowship Program, Brookline, Massachusetts, United States; Northeastern University, Gray, Maine, United States; Northeastern University, Boston, Massachusetts, United States; Northeastern University, Boston, Massachusetts, United States; Northeastern University, Boston, Massachusetts, United States; VA Boston, Boston, Massachusetts, United States; Northeastern University, Boston, Massachusetts, United States

## Abstract

Prevalence of frailty among older sexual and gender minority adults (OSGM) is unknown despite disparities in mental health, medical comorbidities, and physical function. The NIH-funded All of Us Program was launched May 2018 aiming to enroll 1 million US participants focusing on those underrepresented in biomedical research, including OSGM. Using validated methods, we developed an All of Us deficit accumulation frailty index (AoU-FI) consisting of 33-items using baseline survey responses of adults aged 50+. Deficit domains include comorbidities, physical functioning, mental health, cognition, and sensory impairment. AoU-FI was valid if ≤20% of items were missing and ≤70% were comorbidities. OSGM self-identified or had discordance between gender and sex responses. OSGM (n=5,678) and non-OSGM (n=66,325), were similar in age (mean (IQR) = 66.7 (60–73) vs 66.9 (60–74)) but were more diverse (White 78% vs 82%, Black 7.5% vs. 6.5%, Hispanic/Latino 6.9% vs. 5.9%). AoU-FI had an expected gamma distribution across groups. OSGM frailty had a narrower range (0–0.67 vs. 0–0.75) and higher mean of 0.19 (sd=0.11) vs 0.17 (sd=0.1) compared to non-OSGM. To our knowledge, this is the first study of frailty among OSGM. Findings suggest OSGM experience worse frailty, highlighting the need to understand disparities in frailty, identify interventions, and develop policies to support OSGM. Additionally, our novel AoU-FI creates opportunities to apply frailty to diverse participants and types of data from digital health to genomics within the All of Us database.

